# Chemistry in flow systems II

**DOI:** 10.3762/bjoc.7.119

**Published:** 2011-08-02

**Authors:** Andreas Kirschning

**Affiliations:** 1 Leibniz University of Hannover, Schneiderberg 1B, 30167 Hannover, Germany

This is the second Thematic Series on chemistry in flow systems that I have edited for the Beilstein Journal of Organic Chemistry. Is this perhaps too much of a tribute to an enabling technology that will probably never move beyond the status of a trend or a fashion? Have we not already seen this kind of fashionable topic initially promise so much and subsequently not quite meet expectations? For example the impact of “combinatorial chemistry” has turned out to be important, but the field did not live up to the original hype.

No one can predict the future impact of flow chemistry, but it is an enabling technology that was introduced to the laboratories of synthetic organic chemists around ten years ago and has flourished for about half a decade now. We can draw a parallel with microwave-assisted synthesis, for which almost two decades passed until it became a broadly accepted enabling heating technology, and which will continue to have a strong impact on practical organic chemistry. Flow systems have already been implemented in various areas in organic chemistry with very promising results, and the lasting effects will certainly become apparent in the years to come.

I am very pleased to say that this series contains representative examples of fields in organic synthesis where continuous flow conditions have already made a significant impact. In particular I would like to highlight three areas. First of all, we have the application to photochemistry, which has the chance of experiencing a renaissance particularly in an industrial environment. Second, flow chemistry lends itself naturally to the synthesis and direct application of reactive intermediates or reactive reagents, which are difficult to handle in a batch reactor. And third, new heating concepts, including inductive heating, are rendered possible, allowing us to carry out accelerated synthesis under pressure, up to supercritical conditions, but whereby only a small volume of the reaction mixture inside the flow reactor is exposed to these extreme conditions.

You are invited to explore this Thematic Series and you will see contributions from some of the most prominent and creative groups in the world working in the field of flow chemistry. Not surprisingly this series has significantly increased in size since the first Thematic Series "Chemistry in flow systems" published in 2009 [1]. Particularly, I would like to draw your attention to the review on enzyme-mediated synthesis under flow conditions, a topic that has not been covered for a long time in an organic chemistry context. This review clearly illustrates the scope of this enabling technology with far reaching implications even for industrial biotechnology.

I am thankful to all my colleagues who have helped to create this diverse and state of the art flow series. Finally, special thanks are dedicated to the staff of the Beilstein-Institut for their support and professional realization.

Hannover, July 2011

Andreas Kirschning
